# Sleep-wake cycle irregularities in type 2 diabetics

**DOI:** 10.1186/1758-5996-4-18

**Published:** 2012-05-02

**Authors:** Tomoko Nakanishi-Minami, Ken Kishida, Tohru Funahashi, Iichiro Shimomura

**Affiliations:** 1 Department of Metabolic Medicine, Graduate School of Medicine, Osaka University, Suita, Osaka, 565-0871, Japan; 2 Department of Metabolism and Atherosclerosis, Graduate School of Medicine, Osaka University, 2-2 B-5, Yamada-oka, Suita, Osaka, 565-0871, Japan

**Keywords:** Bed-time, Awakening-time, Sleep duration, Diabetes

## Abstract

**Background:**

The incidence of type 2 diabetes mellitus (T2DM) has been increasing in recent years. Sleep loss and circadian rhythm abnormalities are thought to be one of the underlying causes of adverse metabolic health. However, little is known about sleep-wake cycle irregularities in T2DM. The present study compared the bedtime, waking time, and estimated sleep duration between T2DM and non-T2DM subjects.

**Methods:**

The study subjects were 106 consecutive outpatients with lifestyle-related diseases (males/females = 56/50), who answered a questionnaire on sleep status. Subjects were divided into two groups; non-T2DM (n = 32) and T2DM (n = 74) subjects.

**Results:**

T2DM subjects retired to bed on weekdays and holidays significantly later than non-T2DM subjects (23:43 versus 22:52, *p* = 0.0032; 23:45 versus 22:53, *p* = 0.0038, respectively), and woke up significantly later on weekdays and holidays, compared with non-T2DM subjects (06:39 versus 06:08, *p* = 0.0325; 06:58 versus 06:24, *p* = 0.0450, respectively). There was no significant difference in the estimated sleep duration between the two groups. Daytime sleepiness was reported significantly more commonly by T2DM subjects than non-T2DM subjects (*p* = 0.0195).

**Conclusions:**

Sleep-wake cycle irregularities are more common in T2DM subjects than non-T2DM. Confirmation that such irregularity plays a role in the metabolic abnormalities of T2DM requires further investigation in the future.

**Trial registration:**

UMIN 000002998

## Background

The etiology of type 2 diabetes mellitus (T2DM) includes both genetic and environmental factors. The incidence of T2DM has been increasing recently mainly due to changes in lifestyle, such as over-eating, physical inactivity, and sleep deprivation. Sleep is a highly active and dynamic process, and serves immune defense in particular, with an important role in disease resistance [1]. Several studies have reported major differences in the frequency of sleep disturbances between diabetics and non-diabetics [2,3]. Patients with T2DM sleep less than the general population [4]. The recent dramatic increase in the incidence of obesity and diabetes, and the close relationship between sleep cycles and diabetes [5], suggest detrimental deprivation of certain sleep stages [6,7]. The endogenous circadian clock, including the suprachiasmatic nucleus (SCN) in the hypothalamus and peripheral oscillators in vital organs, regulates much of our physiology and behavior across the 24-h day when it is properly aligned with the sleep-wake cycle. The SCN regulates the circadian rhythms in glucose, corticosteroids, leptin and cardiovascular systems through neural and/or humoral signals to the pancreas, liver, adrenal glands, adipose tissues and heart [8]. Shift work is associated with chronic misalignment between the endogenous circadian timing system and behavioral cycles, including sleep-wake and fasting-feeding cycles [9,10]. Therefore, health problems are not uncommon in shift workers [11]. Chronic circadian misalignment has been proposed to correlate with metabolic and cardiovascular dysfunction [12-16]. However, whether disruption of the sleep-wake pattern, i.e., sleep-wake cycle irregularity, relates to T2DM remains to be elucidated.

The present study compared the sleeping and waking times in subjects with and without T2DM.

## Methods

### Participants

Subjects were recruited from consecutive Japanese outpatients with metabolic lifestyle-related diseases (e.g., hypertension, dyslipidemia, diabetes and/or gout/hyperurecemia), who visited the Department of Metabolic Medicine, Osaka University Hospital, between October and December 2011, and answered a questionnaire on sleep status. The exclusion criteria included pregnant women, nursing mothers, and nighttime and/or shift workers. Disease-related exclusion criteria included pituitary diseases, mental disorders, and malignant diseases. The study subjects were 106 consecutive outpatients (males/females = 56/50). The study was approved by the Medical Ethics Committee of Osaka University. All participants were Japanese and each gave a written informed consent. This study (The Endocrine-Metabolic Disease and Sleep Apnea Syndrome Study) is registered under number UMIN 000002998 https://upload.umin.ac.jp/cgi-open-bin/ctr/ctr.cgi?function=brows&action=brows&type=summary&recptno=R000003635&language=E.

### Self-questionnaire on sleep habits

The questionnaire on sleep patterns consisted of the following 8 questions: bedtime on weekdays and holidays (at half-hour intervals); waking time on weekdays and holidays (at half-hour intervals); arousal (yes or no); daytime sleepiness (yes or no); insomnia due to work (yes or no); insomnia due to mental stress (yes or no). Sleep duration (hours) = waking time – bedtime.

### Anthropometric data and laboratory tests

Height (cm), weight (kg), and body mass index (BMI) in kg/m^2^ were measured in the standing position. Systolic- and diastolic- blood pressures were measured with a standard sphygmomanometer after at least 5-min rest. After overnight fasting, venous blood samples were collected while the subject was in the supine position for measurements of blood glucose, glycoalbumin, hemoglobin A1c (HbA1c), triglyceride, high-density lipoprotein-cholesterol (HDL-C), uric acid, and creatinine. Low-density lipoprotein-cholesterol (LDL-C) was calculated with the Friedewald equation. The value for HbA1c (%) was estimated as National Glycohemoglobin Standardization Program (NGSP) equivalent value (%), calculated by the formula HbA1c (%) = HbA1c (Japan Diabetes Society [JDS], %) + 0.4%.

Diabetes mellitus was defined according to the criteria of the World Health Organization and/or current treatment for diabetes mellitus (sulfonyl ureas/biguanides/α-glucosidase inhibitors/pioglitazone/ dipeptidyl peptidase-4 inhibitors/glinides/insulin/glucagon-like peptide-1 analogue, n = 28/27/23/10/14/2/17/2). Diabetic retinopathy, nephropathy and peripheral neuropathy were diagnosed as reported previously [17]. Dyslipidemia was defined as total cholesterol of ≥220 mg/dL, triglyceride ≥150 mg/dL, HDL-C <40 mg/dL, and/or current treatment for dyslipidemia (statins/fibrates/ezetimibe; n = 40/4/5). Hypertension was defined as systolic blood pressure ≥140 mmHg, diastolic blood pressure ≥90 mmHg, or current treatment for hypertension (calcium channel antagonists/angiotensin converting enzyme inhibitors/angiotensin receptor blockers/β-blockers/α-blockers/diuretics/ direct renin inhibitor; n = 35/5/36/8/2/9/2).

### Statistical analysis

All values were expressed as mean±SEM. In all cases, a *p* value <0.05 denoted the presence of a statistically significant difference. Differences between two groups were compared by unpaired Student’s *t*-test. Differences in frequencies were compared by the χ^2^ test. All analyses were performed with the JMP Statistical Discovery Software 9.0 (SAS Institute, Cary, NC).

## Results

### Characteristics of T2DM and non-T2DM subjects

Subjects with lifestyle-related diseases were divided into two groups; with T2DM and non-T2DM. The baseline characteristics of the two groups are listed in Table 1. T2DM subjects had significantly higher BMI, lower serum HDL-C levels, higher prevalence of hypertension, than non-T2DM subjects.

**Table 1:** Baseline characteristics of subjects with type 2 diabetes mellitus and control subjects (n = 106)

	Control subjects (n = 32)	T2DM subjects (n = 74)	p value
Gender, male/female	19/13	37/37	0.4041
Age, years	62 ± 1 (39-83)	66 ± 1 (36-84)	0.5349
Job type (non/employee/individual proprietor/homemaker/others)	5/14/1/11/1	17/25/1/27/4	
Body mass index, kg/m^2^	22.7 ± 0.7 (13.9-30.8)	24.7 ± 0.5 (17.8-34.5)	**0.0153**
Blood glucose, mg/dL	94 ± 3 (53-113)	128 ± 74 (52-252)	**<0.001**
Glycoalbumin, %	14.9 ± 1.0 (12.9-16.0)	19.8 ± 0.6 (12.5-33.3)	**0.0243**
HbA1c (NGSP), %	5.9 ± 0.1 (5.4-6.3)	7.0 ± 0.1 (5.6-14.4)	**0.0001**
Systolic blood pressure, mmHg	138 ± 23 (98-174)	137 ± 2 (101-182)	0.9149
Diastolic blood pressure, mmHg	81 ± 2 (65-97)	80 ± 1 (49-105)	0.7800
Triglyceride, mg/dL	175 ± 41 (44-1231)	138 ± 10 (34-471)	0.8158
High-density lipoprotein cholesterol, mg/dL	62 ± 23 (31-121)	53 ± 2 (17-103)	**0.0079**
Low-density lipoprotein cholesterol, mg/dL	117 ± 5 (80-196)	112 ± 4 (64-208)	0.3333
Uric acid, mg/dL	5.5 ± 0.2 (2.8-7.8)	5.5 ± 0.2 (2.7-9.4)	0.9827
Creatinine, mg/dL	0.70 ± 0.02 (0.46-1.15)	0.86 ± 0.05 (0.44-2.83)	0.0798
Diabetic neuropathy	-	n = 15	
Diabetic retinopathy (NDR/SDR/PDR)	-	n = 56/6/12	
Diabetic nephropathy (stage I/II/III/IV)	-	n = 57/9/3/5	
Drugs for diabetes (medication/insulin)	-	n = 57/17	
Hypertension (under medications)	n = 18 (n = 11)	n = 60 (n = 46)	**0.0151**
Dyslipidemia (under medications)	n = 21 (n = 13)	n = 48 (n = 34)	0.8253
Insomnia, under medications	n = 6	n = 11	0.7736

Data are mean ± SEM or n (range). Significant level was set at p value <0.05 (bold type). T2DM: type 2 diabetes mellitus, NDR: non-diabetic retinopathy, SDR: simple diabetic retinopathy, PDR: proliferative diabetic retinopathy.

### Bedtime, waking time, and sleep duration

**Figure 1:**
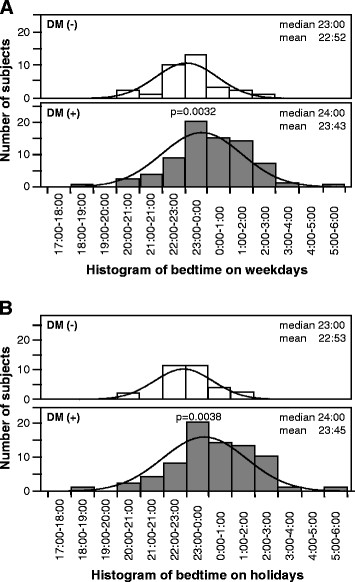
Histograms of the numbers of subjects with type 2 diabetes mellitus (DM+) and non-diabetic subjects (DM-) for each bedtime on (A) weekdays and (B) holidays.

Figure 1 is a histogram of reported bedtime on weekdays and holidays in T2DM and non-T2DM subjects. The bedtime on weekends and holidays was significantly later in T2DM subjects, compared to non-T2DM subjects (23:43±0:12 versus 22:52±0:13, p = 0.0032, Figure 1A; 23:45±0:12 versus 22:53±0:13, p = 0.0038, Figure 1B).

**Figure 2:**
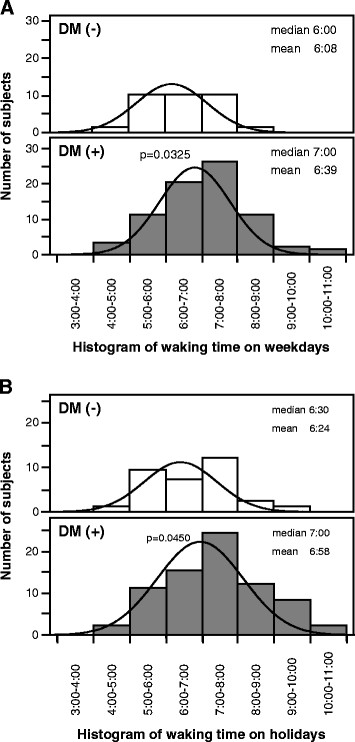
Histograms of the numbers of subjects with type 2 diabetes mellitus (DM+) and non-diabetic subjects (DM-) for each waking time on (A) weekdays and holidays (B).

Figure 2 is a histogram of waking time on weekdays and holidays in T2DM and non-T2DM subjects. The waking time was significantly later in T2DM subjects on weekends and holidays, compared to non-T2DM subjects (06:39±0:08 versus 06:08±0:02, p = 0.0325, Figure 2A; 06:58±0:08 versus 06:24±0:12, p = 0.0450, Figure 2B).

**Figure 3:**
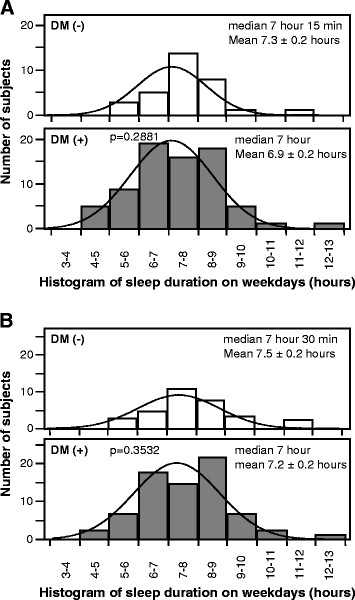
Histograms of the numbers of subjects with type 2 diabetes mellitus (DM+) and non-diabetic subjects (DM-) for different sleep durations on (A) weekdays and (B) holidays.

There was no significant difference in the estimated sleep duration on weekdays and holidays between the two groups (Figure 3).

### Correlation between sleep-wake parameters and HbA1c

**Figure 4:**
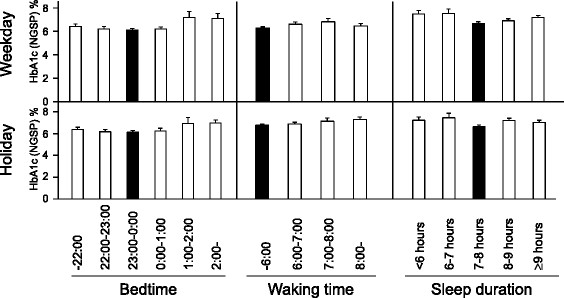
**Mean HbA1c levels at various bed and waking times, and according to sleep duration on weekdays (top) and holidays (bottom).** Data are mean±SEM.

In bedtime analysis, the lowest HbA1c levels were 6.5±0.1% and 6.6±0.1% recorded at bedtime 23:00–00:00 on weekdays and on holidays, respectively (Figure 4 left, solid box). In waking time analysis, the lowest HbA1c levels were 6.7±0.1% and 6.6±0.1% in waking time <06:00 on weekdays and holidays, respectively (Figure 4 middle, solid box). In sleep duration analysis, the lowest HbA1c levels were 6.4±0.1% and 6.4±0.2% in subjects who slept for 7–8 h on weekdays and holidays, respectively (Figure 4 right, solid box).

### Incidence of sleep-related problems

**Figure 5:**
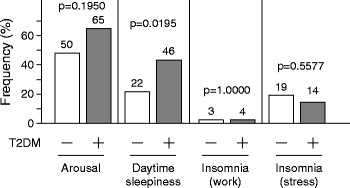
Comparisons of the frequency of reported arousals, daytime sleepiness, insomnia (due to work) and insomnia (due to mental stress) in subjects with type 2 diabetes mellitus (DM+) and non-diabetic subjects (DM-).

The prevalence of daytime sleepiness was significantly higher in T2DM subjects than in non-T2DM subjects (46% versus 22%, p = 0.0195, Figure 5). However, there were no significant differences in the frequency of arousals at night or percentages of subjects with work- and mental stress-related insomnia between the two groups.

## Discussion

The major findings of the present study were that T2DM subjects retired to bed later and woke up later, and suffered from daytime sleepiness, compared with non-T2DM subjects. The 2006 Ministry of Internal Affairs and Communications (Sōmu-shō) Survey on Time Use and Leisure Activities indicates that the average bedtime of 66,428 Japanese (males/females; 31,520/34,908) is 23:16 on weekdays, with waking time on weekdays of 06:39. Comparison of the above data and the present findings confirm that the Japanese T2DM subjects tended to retire to bed relatively later than the rest of the population.

The circadian system is linked to various processes that control both sleep and metabolism. The sleep-wake cycle and fasting-feeding behavior are considered to be regulated by the circadian clock [14-16]. Experimental models of the clock gene have demonstrated the development of metabolic disorders, such as obesity and T2DM, after disruption of the circadian rhythms [18-22]. However, whether abnormal glucose metabolism has any impact on the circadian rhythm remains unclear. A vicious cycle may ensue in the disrupted glucose metabolic pathways, leading to lengthening of the circadian oscillation abnormality. Scheer et al. showed that diurnal variation of neurohumoral factors, such as leptin and cortisol, plays a role in behavioral cycles (fasting/feeding and sleep/wake cycles) [13]. Further studies are required to investigate circadian variation of neurohumoral factors in blood. In this regard, the present work is a cross-sectional study, making it difficult to establish a cause-effect relationship. Sleep management, e.g., lifestyle modification by trained medical staff and use of exogenous melatonin as a potential chronotherapeutic agent, may be important to improve the disrupted cardiometabolic system in T2DM subjects with sleep-wake irregularities. Further prospective interventional studies should be conducted in the future to investigate this relationship.

Patients with T2DM sleep less than the general population [4]. A gradual decrease in self-reported sleep duration seems to have occurred with the dramatic recent increase in the incidence of obesity and diabetes, including a close relationship between sleep cycle and diabetes [23-25]. However, the present study found no significant difference in the sleep duration between the diabetics and non-diabetic subjects (Figure 3). However, records of bedtime and waking time were based in the present study on information provided by the subjects, and therefore the sleep duration may be inaccurate.

Previous studies reported that both short (<5 h) and long (>9 h) sleepers as well as those with sleep loss, are at greater risk for glucose intolerance and T2DM [26-33]. The present study found that late bedtime (>1:00) sleepers as well as short and long sleepers had elevated HbA1c (Figure 4). Taken together, irregular sleep-wake patterns as well as short and long sleep may enhance glucose dysmetabolism.

## Conclusion

The present study demonstrated late bedtime and late wake-up time, with daytime sleepiness in T2DM subjects, compared with non-T2DM subjects. Early retirement to sleep and early morning rise seems potentially simple and useful therapeutic target for diabetes.

### Study limitations

There are several limitations to this study. First, all outpatients in this study were Japanese and any differences from other ethnicities are unknown. Second, there is bias in single center studies. Our study included only a limited number of subjects and further multi-center studies of larger samples should be conducted in the future. Finally, the percentage of non-employees among diabetics was 23.0% (n = 17/74), compared to those among non-diabetics (n = 5/32, 15.6%) (Table 1), although there was no significant difference between two groups (*p* = 0.4824). Diabetics often perform the capillary blood sugar self-test before go to bed. These points may influence the results.

## Abbreviations

HbA1c = hemoglobin A1c; HDL-C = high-density lipoprotein-cholesterol; LDL-C = low-density lipoprotein-cholesterol; T2DM = type 2 diabetes mellitus.

## Competing interests

Ken Kishida and Tohru Funahashi are members of the “Department of Metabolism and Atherosclerosis”, a sponsored course endowed by Kowa Co. Ltd. and a company researcher is dispatched to the course. All other authors declare no competing interests.

## Authors’ contributions

TN-M and KK analyzed the data and wrote the manuscript. KK also recruited and collected data from the patients, and participated in the concept and design of the study, interpretation of data and reviewed/edited the manuscript. TF and IS contributed to the discussion and wrote the manuscript. All authors read and approved the final version of the manuscript.
